# Supplementary material for: Metabolism of the Genus Guyparkeria Revealed by Pangenome Analysis
Source: Microorganisms. 2022 Mar 28;10(4):724. doi: 10.3390/microorganisms10040724 (PMC9032823; doi:10.3390/microorganisms10040724)
Supplement: Supplementary file 1 [file microorganisms-10-00724-s001.zip › microorganisms-1600547-Figure S1.pdf]

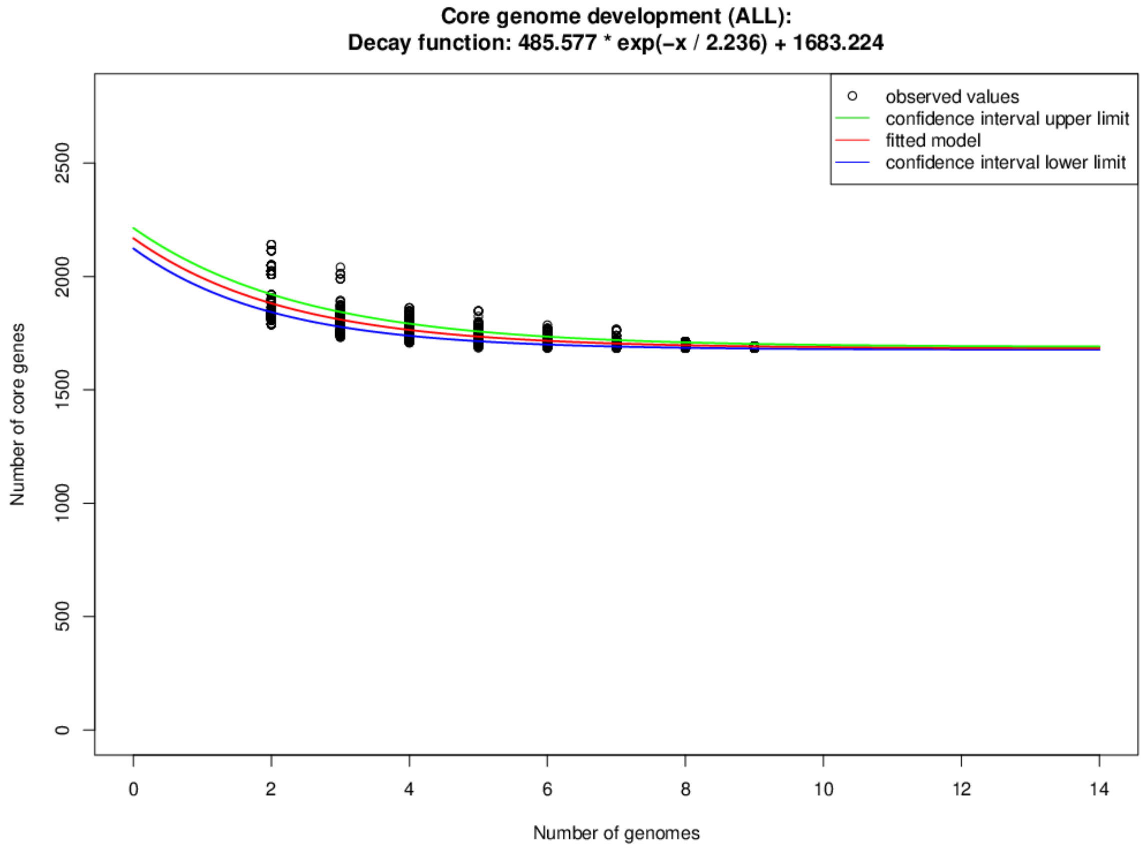

Figure S1. Core genome development plot for 9 *Guyparkeria* genomes (with *Halothiobacillus* sp. S21.Bin061 excluded).
